# Supplementary material for: Modeling the zebrafish gut microbiome’s resistance and sensitivity to climate change and parasite infection
Source: Front Microbiomes. 2025 Jul 22;4:1605168. doi: 10.3389/frmbi.2025.1605168 (PMC12993498; doi:10.3389/frmbi.2025.1605168)
Supplement: Supplementary file 1 [file DataSheet1.zip › Supplemental_Figures__Frontiers_v1.docx]

**Modelling the zebrafish gut microbiome's resistance and sensitivity to climate change and infection**

Michael J. Sieler Jr.^1^, Colleen E. Al-Samarrie^1^, Kristin D. Kasschau^1^, Mike L. Kent^1,2,3^, Thomas J. Sharpton^1,3^

**Supplemental Figures**

Table of Contents

[2) Water temperature shapes gut microbiome structure 3](#_Toc193465253)

[S2A) 3](#_Toc193465254)

[S2B) 3](#_Toc193465255)

[S2C) 3](#_Toc193465256)

[S2D) 4](#_Toc193465257)

[3) Infection burden is highest in fish reared at lower water temperatures 5](#_Toc193465258)

[S3A) 5](#_Toc193465259)

[S3B) 6](#_Toc193465260)

[S3B.1) 6](#_Toc193465261)

[S3B.2) 6](#_Toc193465262)

[S3C) 7](#_Toc193465263)

[4) Gut microbiome response to parasite exposure varies across water temperature 8](#_Toc193465264)

[S4A) 8](#_Toc193465265)

[S4B) 8](#_Toc193465266)

[S4C) 9](#_Toc193465267)

[S4D) 9](#_Toc193465268)

[5) Gut microbiome response has a non-linear relationship with infection burden 10](#_Toc193465269)

[S5A) 10](#_Toc193465270)

[S5B) 10](#_Toc193465271)

[S5C) 11](#_Toc193465272)

[S5D) 11](#_Toc193465273)

[S5D.1) 11](#_Toc193465274)

[S5D.2) 12](#_Toc193465275)

[S5D.3) 12](#_Toc193465276)

[S5D.4) 13](#_Toc193465277)

[6) Parasite exposure exacerbates water temperature differences in gut microbiome structure 14](#_Toc193465278)

[S6A) 14](#_Toc193465279)

[S6B) 14](#_Toc193465280)

[S6C) 15](#_Toc193465281)

[S6D) 15](#_Toc193465282)

[7) Gut microbial abundance is significantly associated with environmental conditions and stressors Error! Bookmark not defined.](#_Toc193465283)

# 2) Water temperature shapes gut microbiome structure

## S2A)


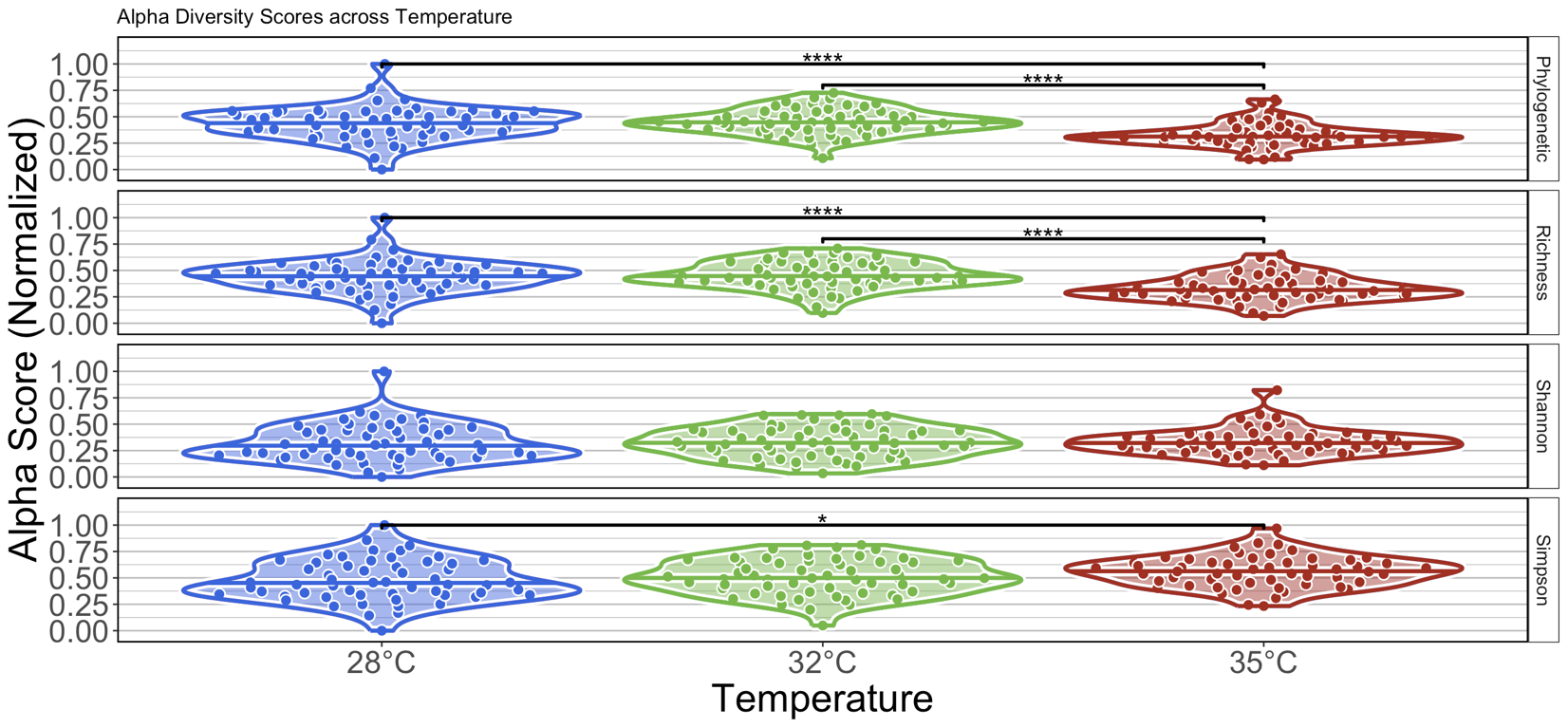


## S2B)

| 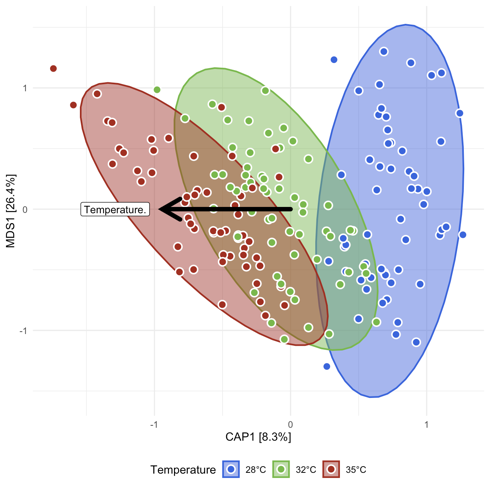 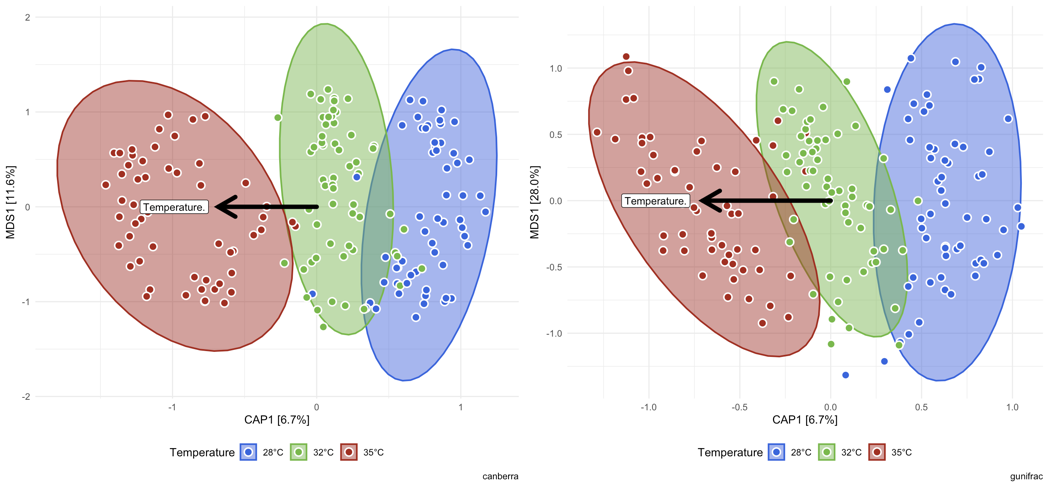 |
| --- |

## S2C)

| 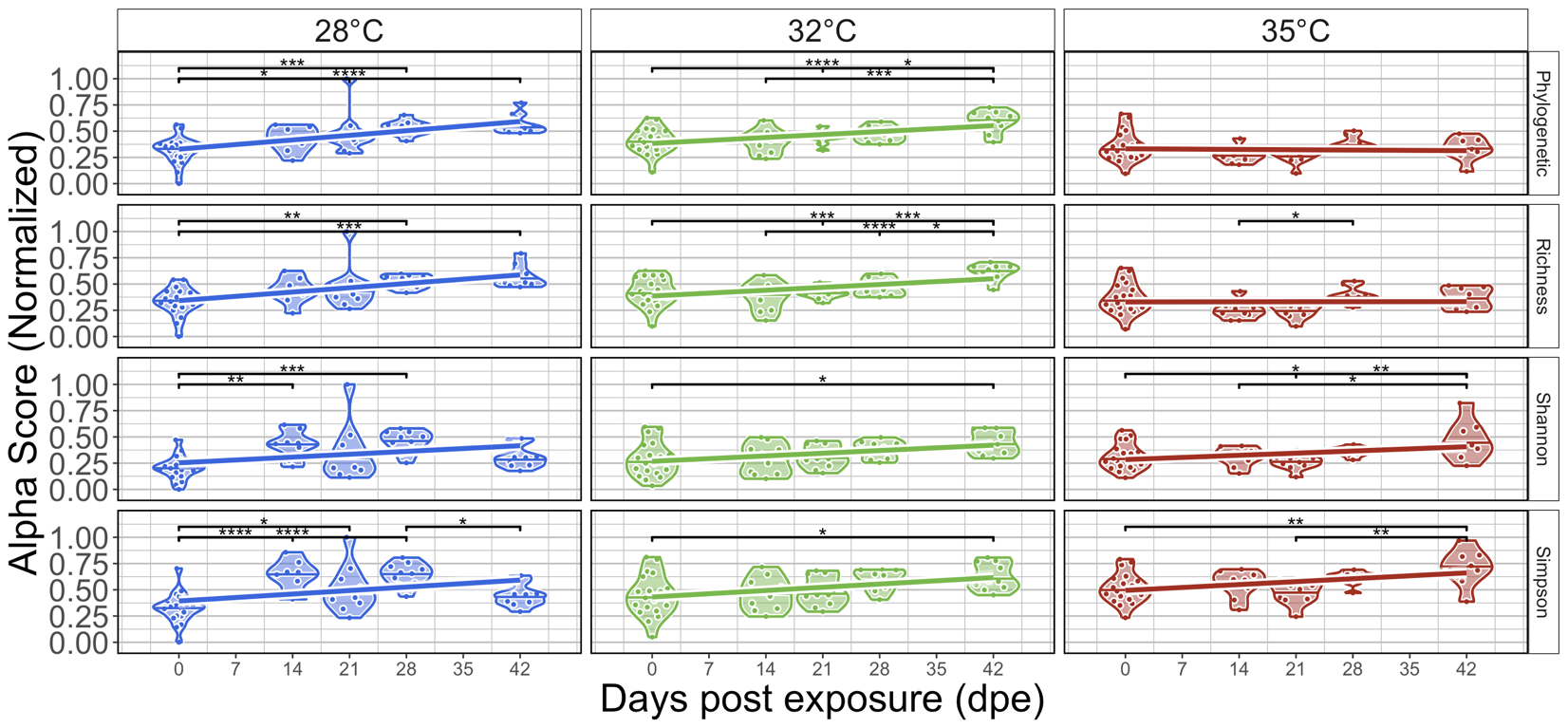 |
| --- |

## S2D)

| 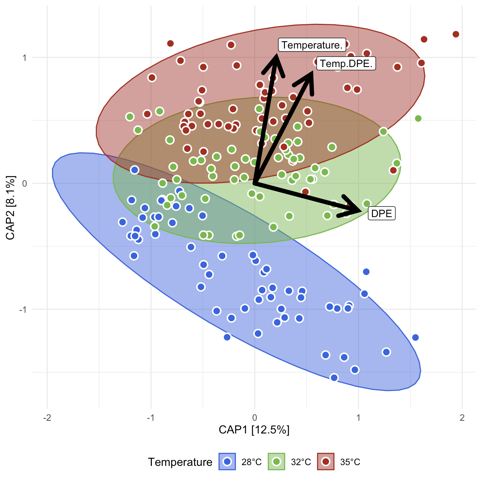 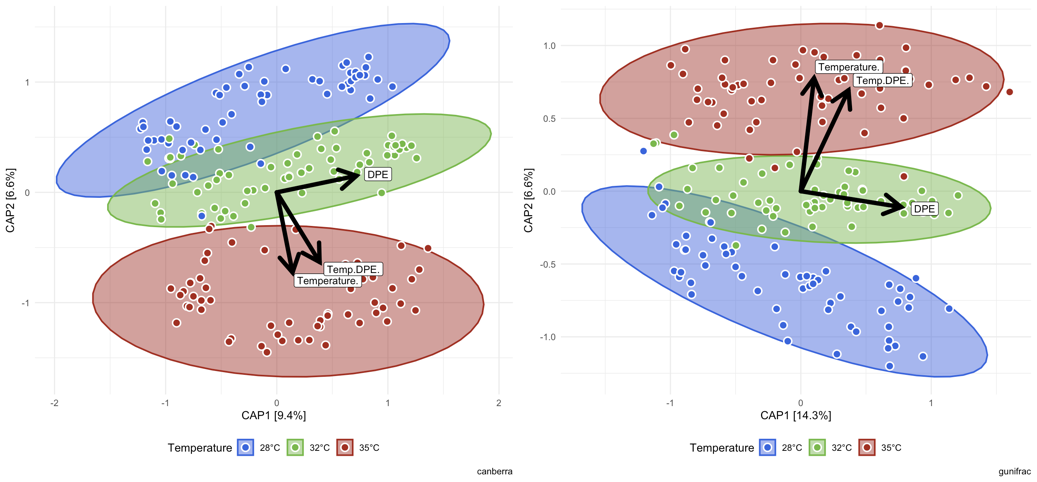  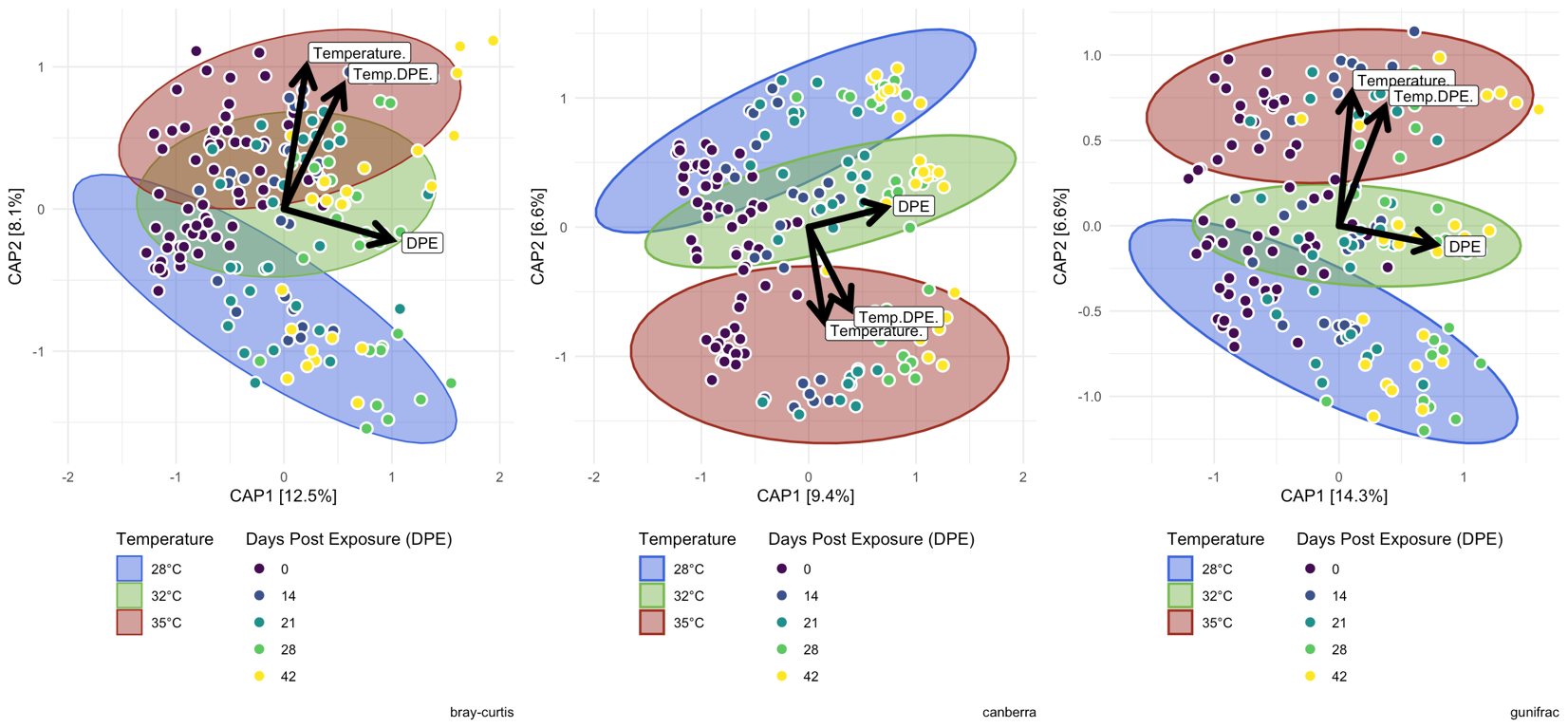 |
| --- |

# 3) Infection burden is highest in fish reared at lower water temperatures

## S3A)

### S3A.1)

| 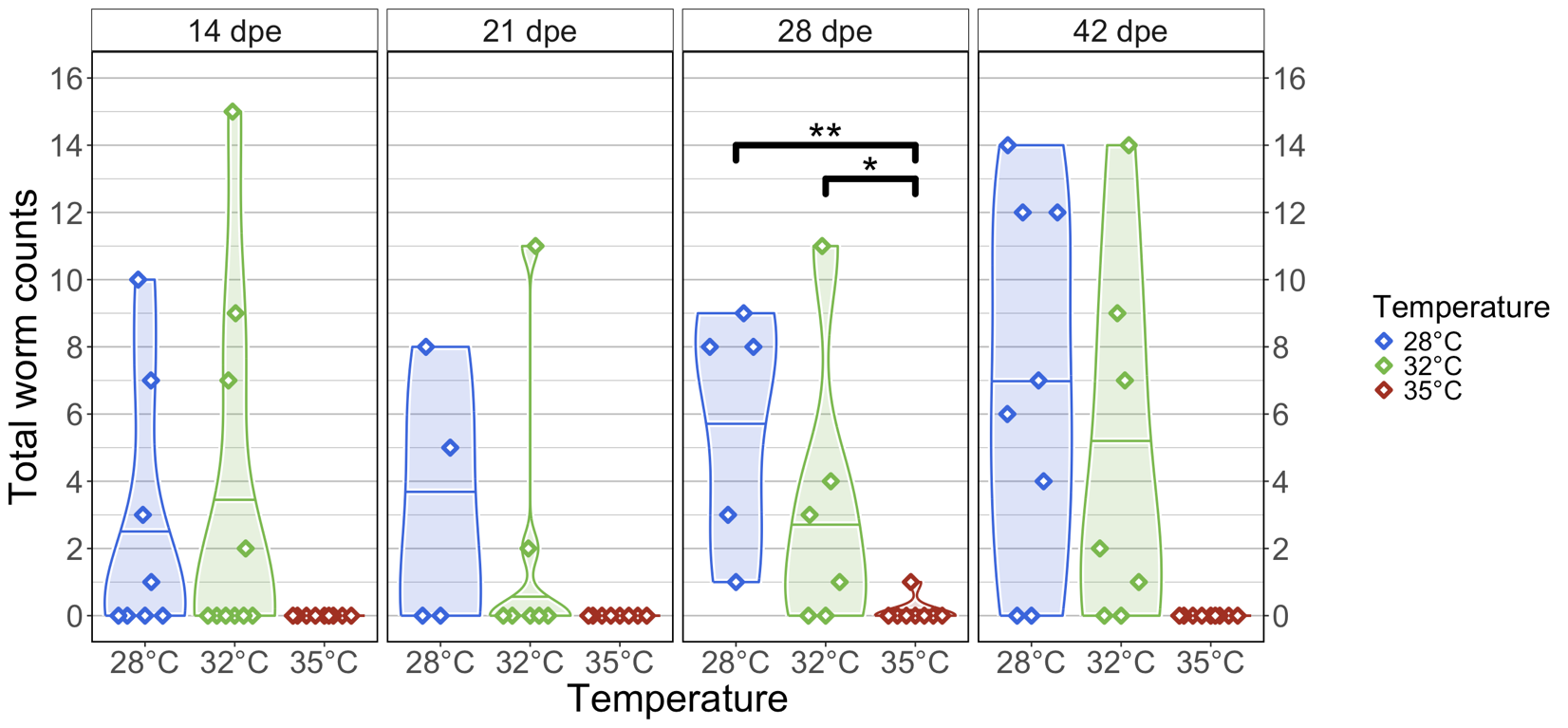 |
| --- |

### S3A.2)

| 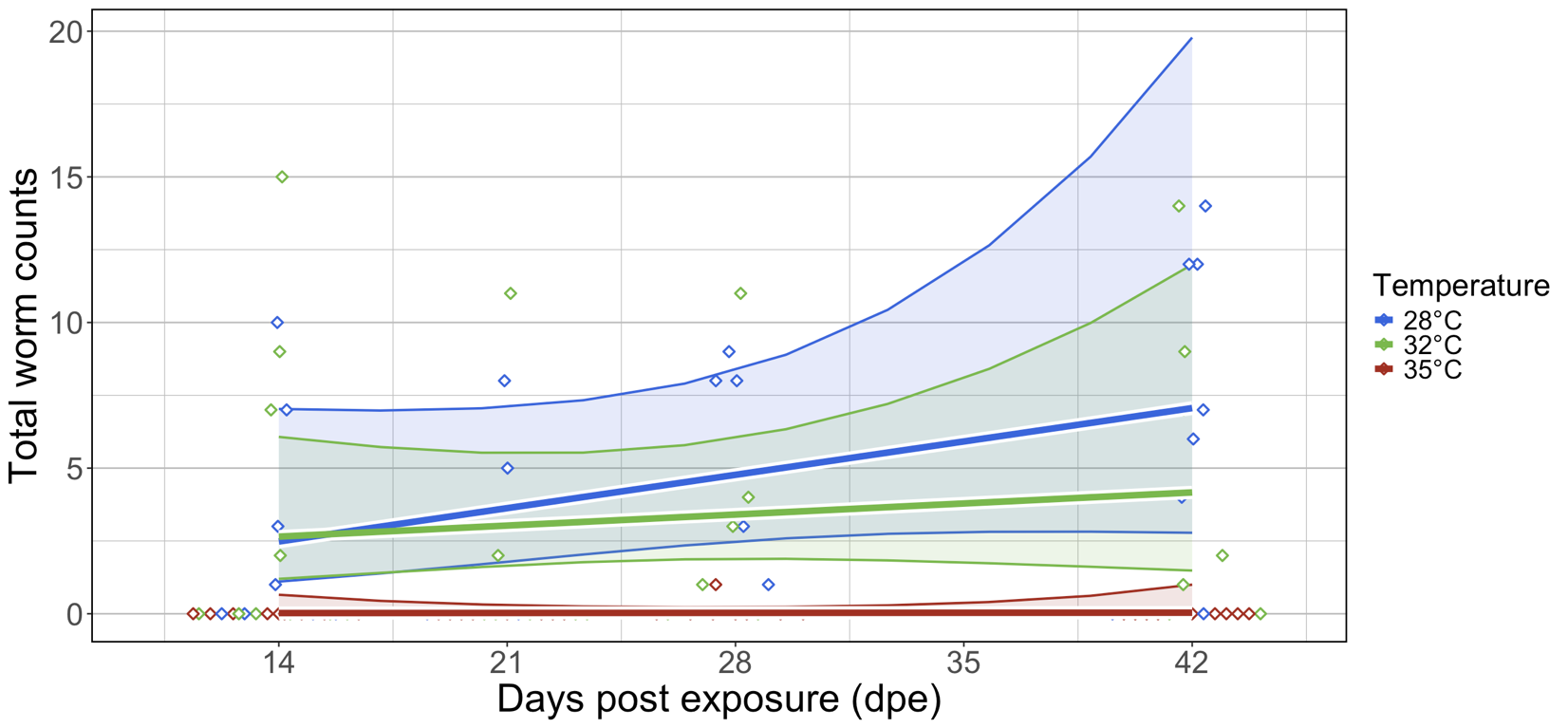 |
| --- |

## S3B)

| 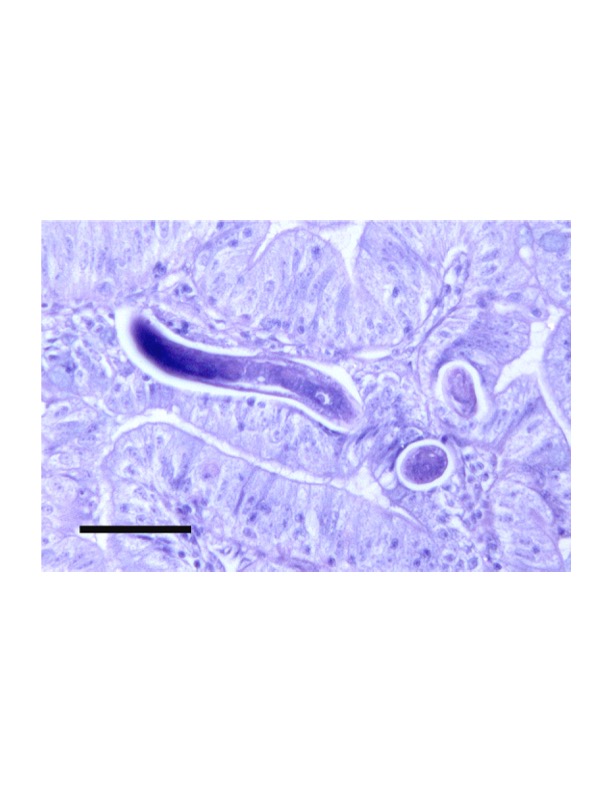 |
| --- |

## S3C)

| 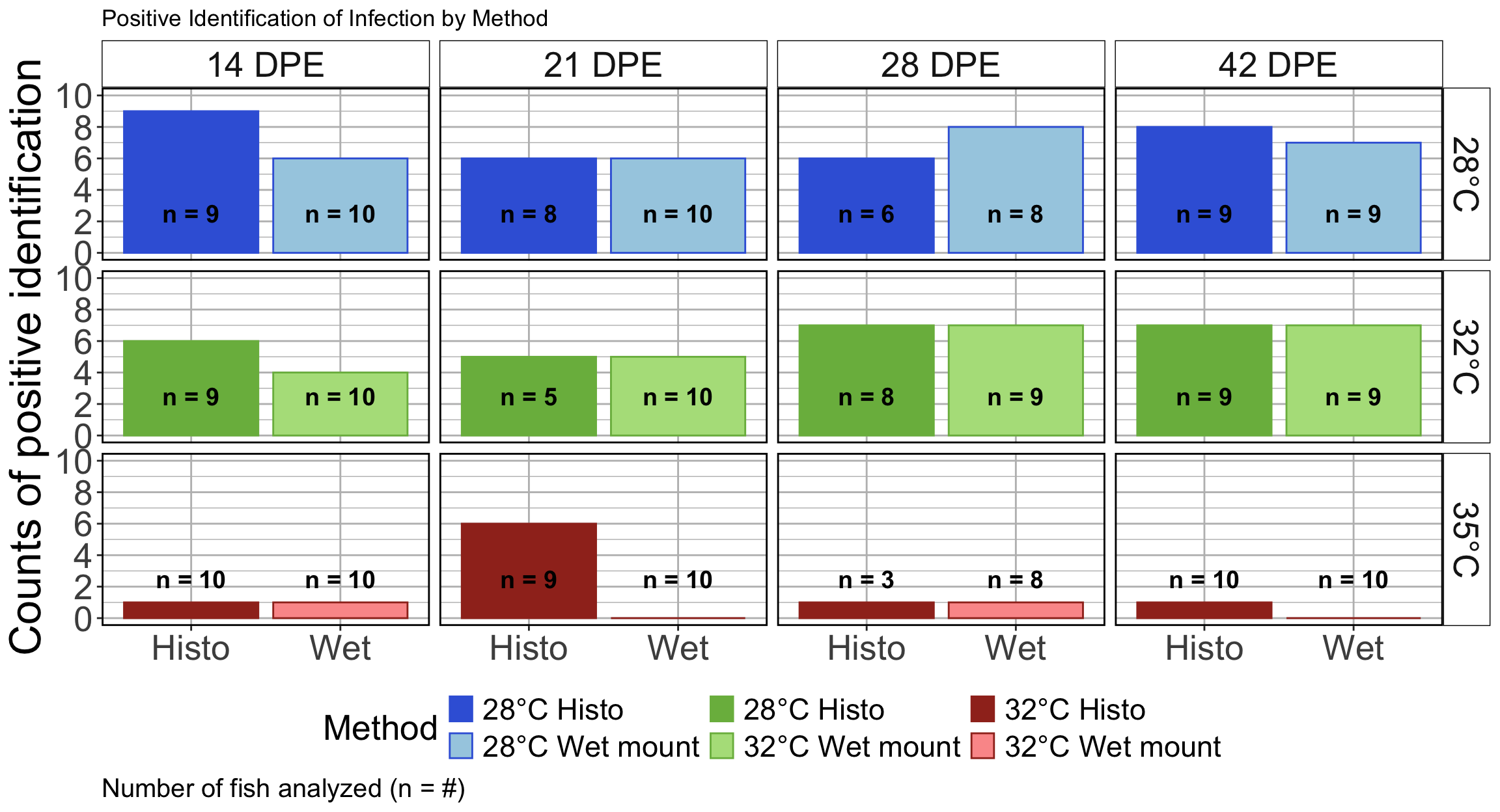 |
| --- |

# 4) Gut microbiome response to parasite exposure varies across water temperature

## S4A)

| 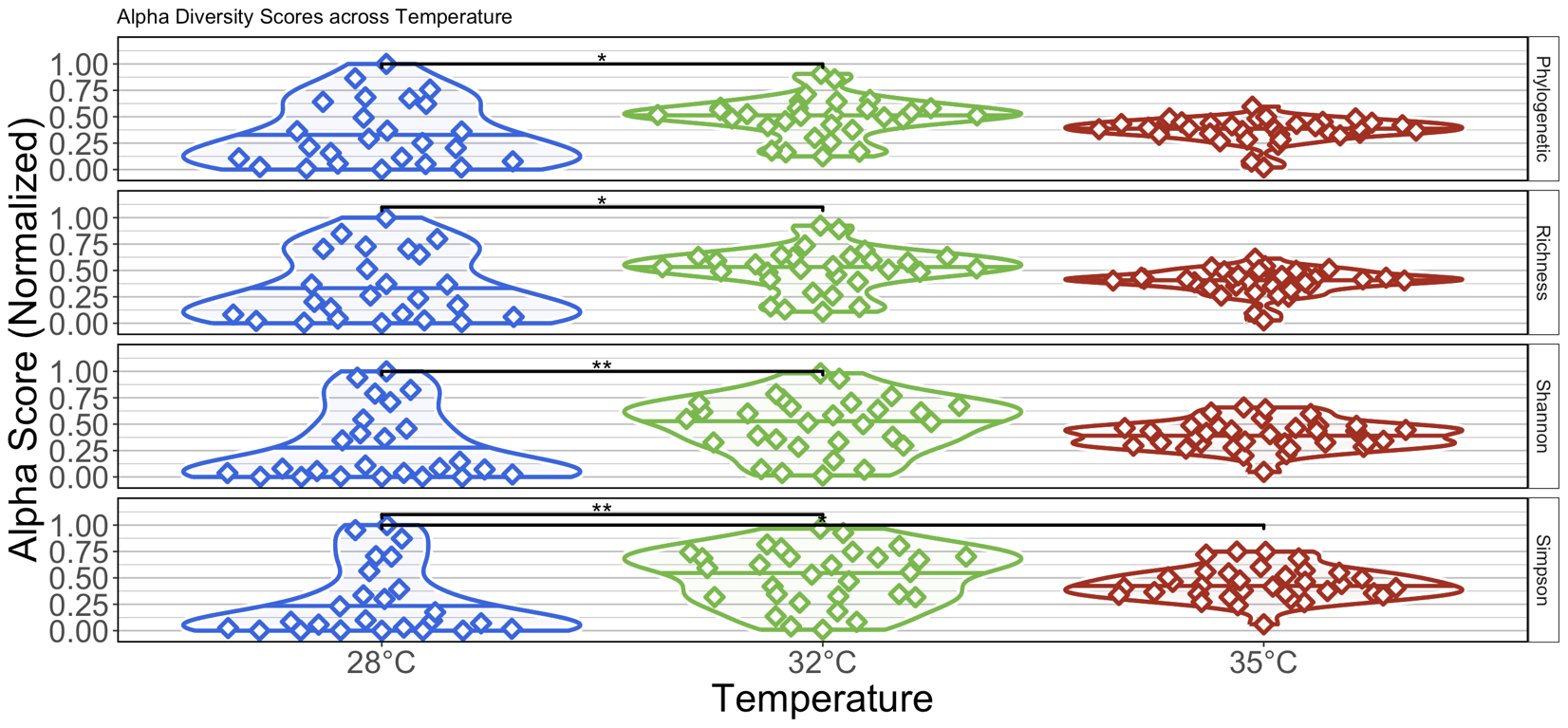 |
| --- |

## S4B)

| 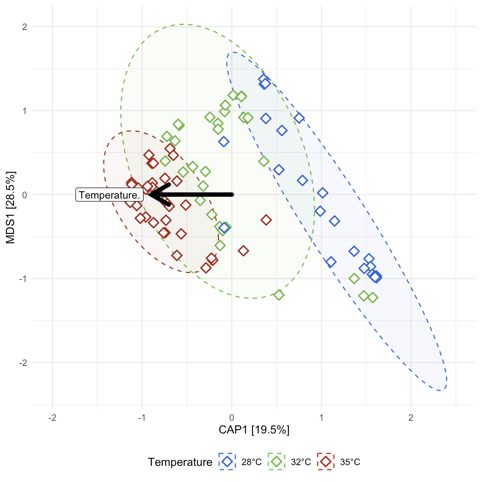 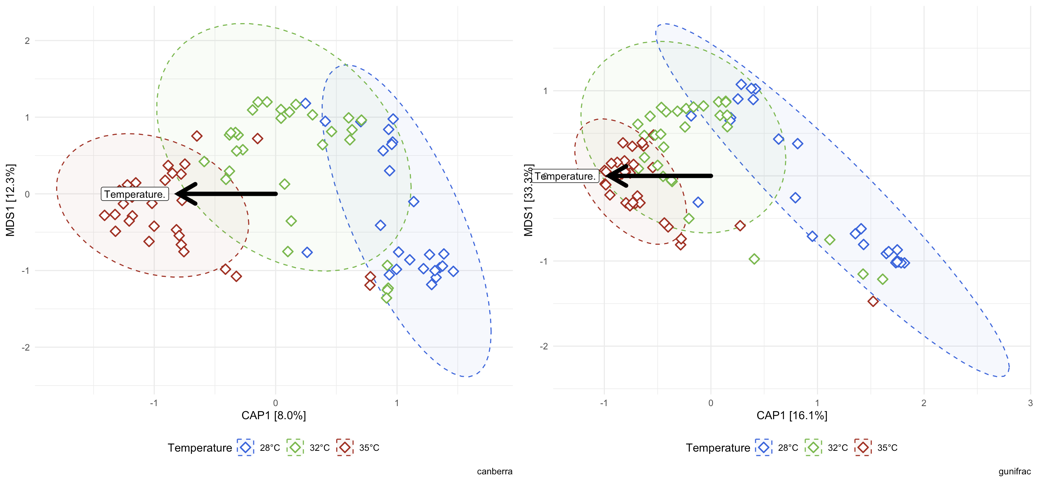 |
| --- |

## S4C)

| 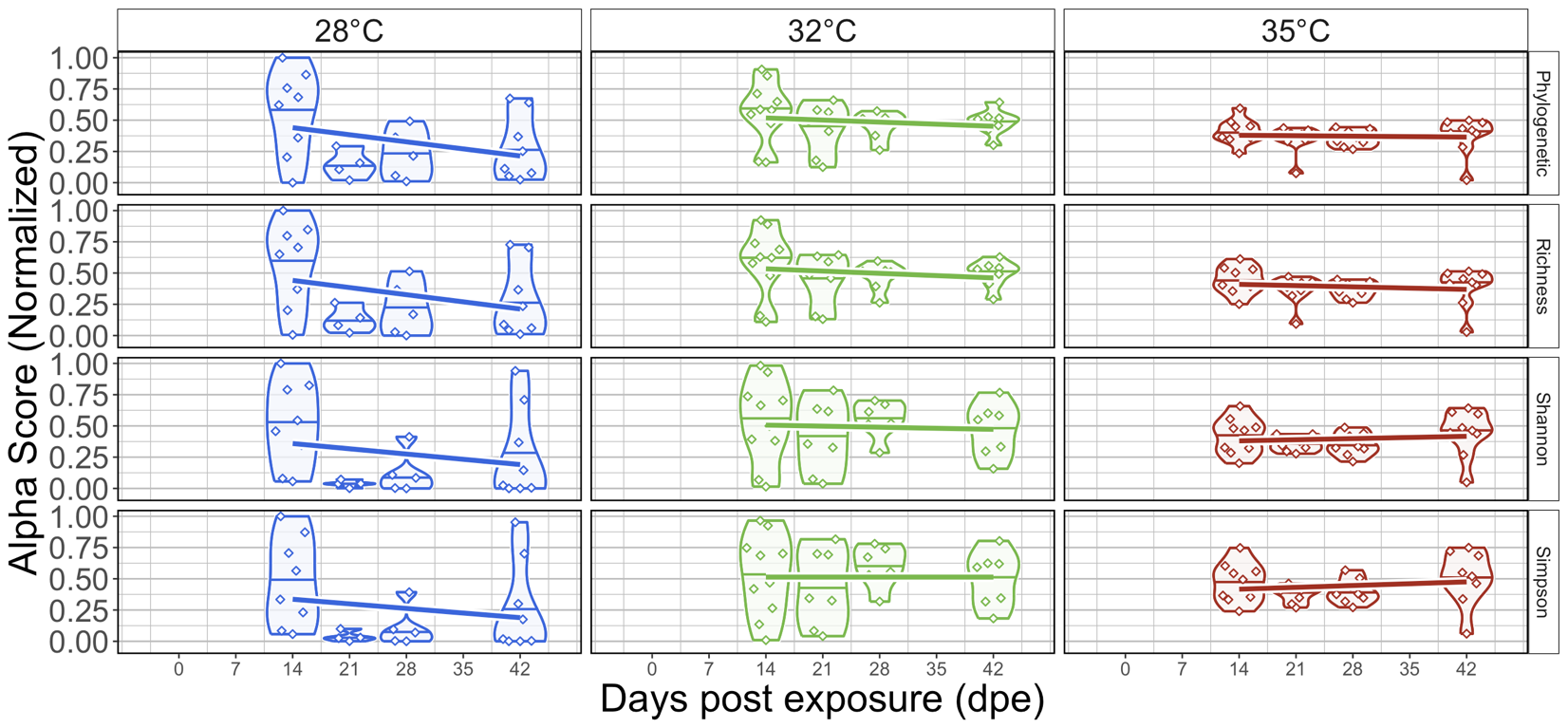 |
| --- |

## S4D)

| 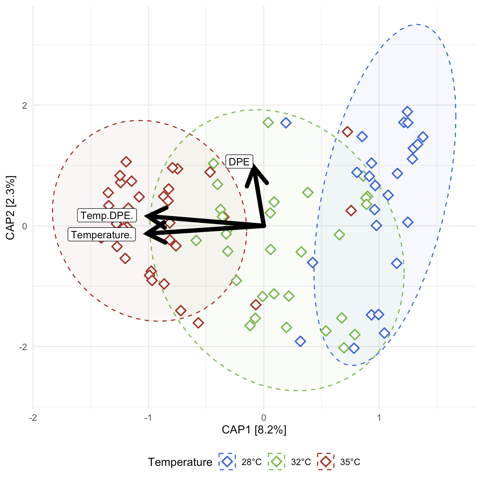 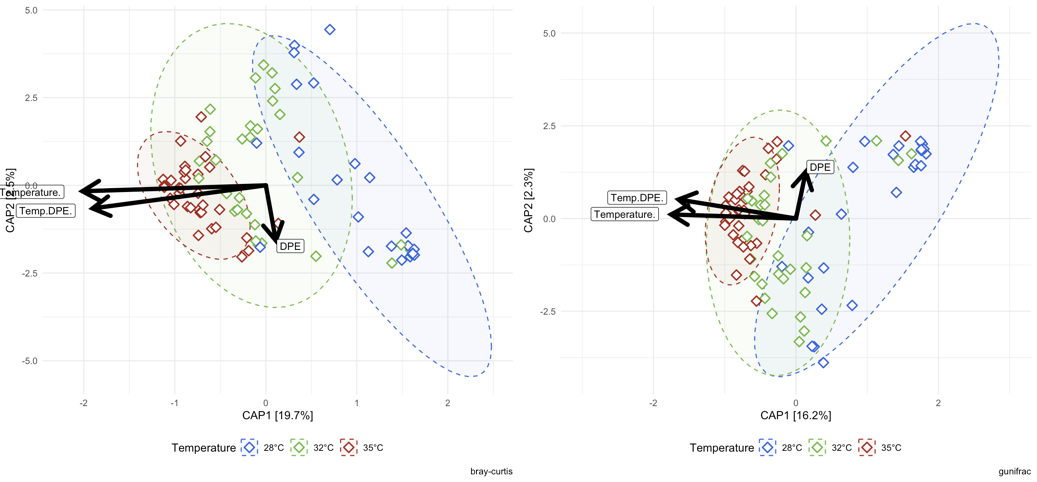  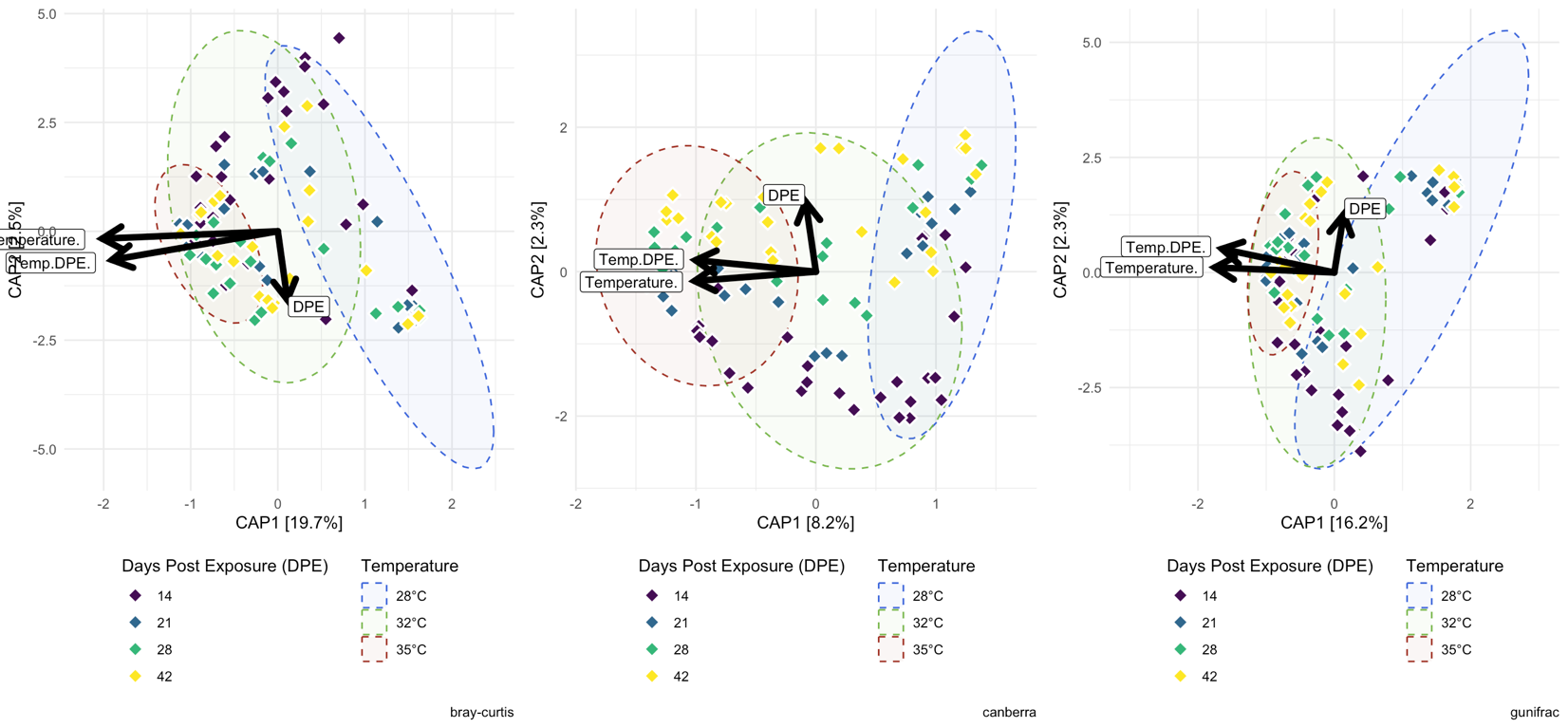 |
| --- |

# 5) Gut microbiome response has a non-linear relationship with infection burden

## S5A)

| 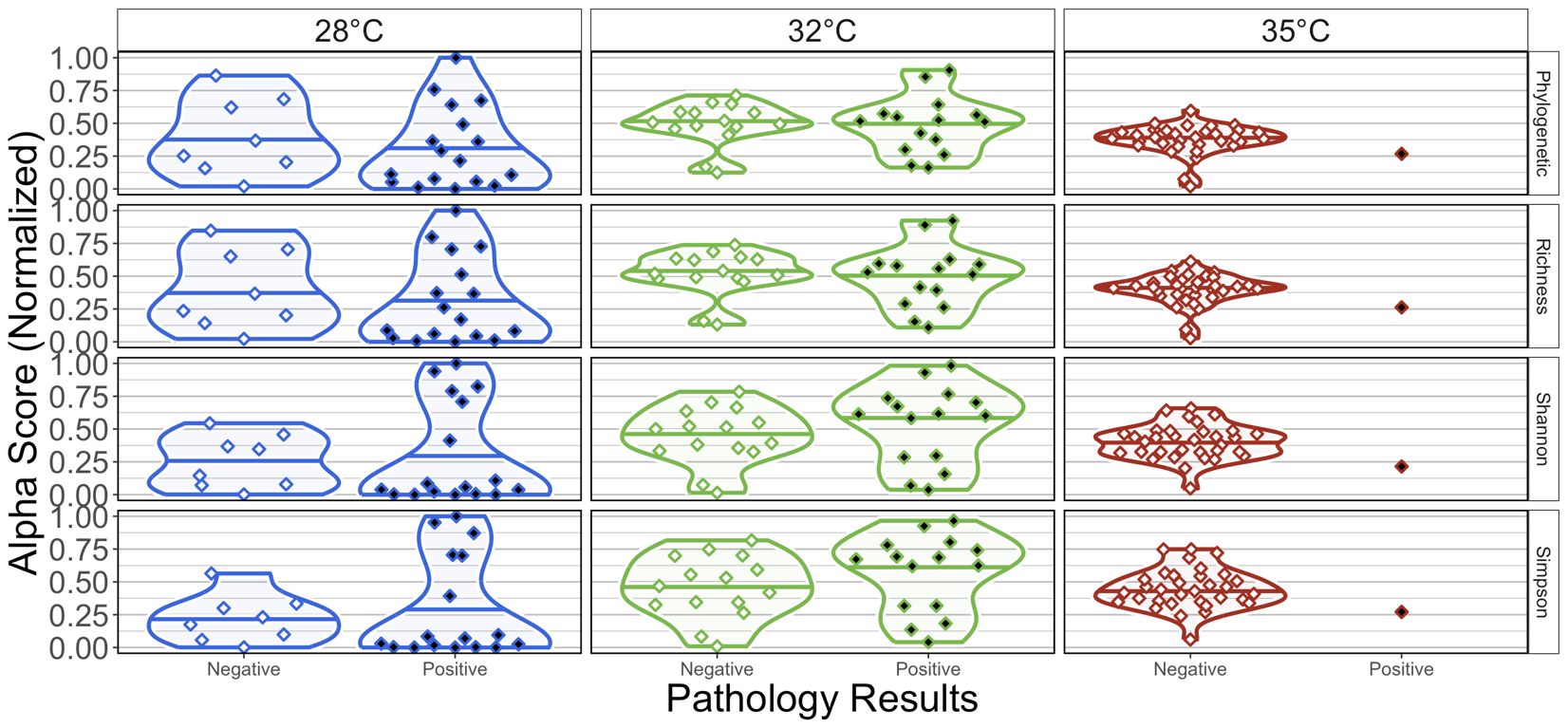 |
| --- |

## S5B)

| 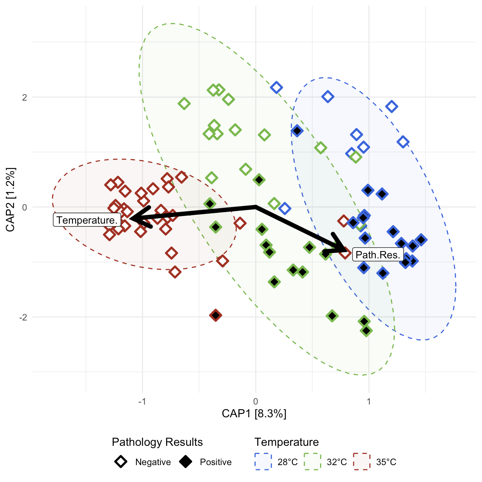 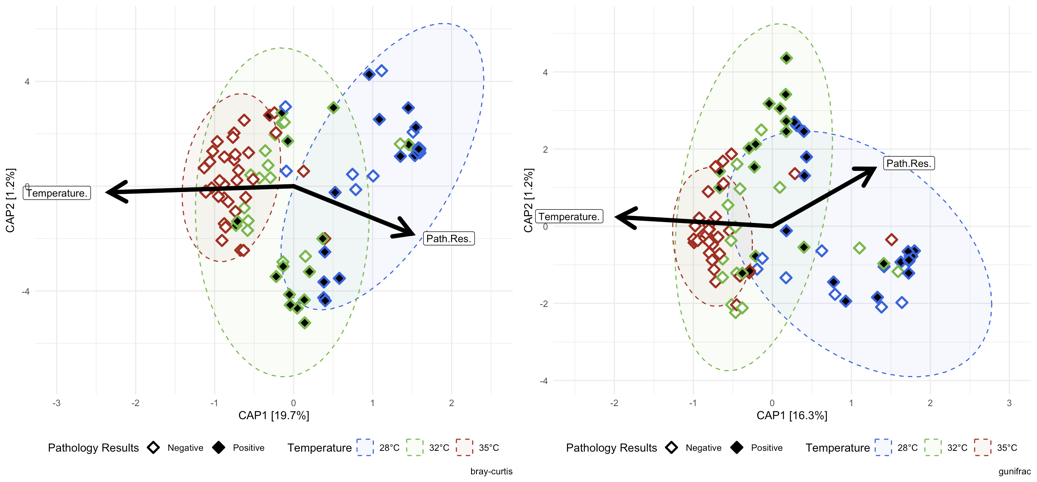 |
| --- |

## S5C)

| 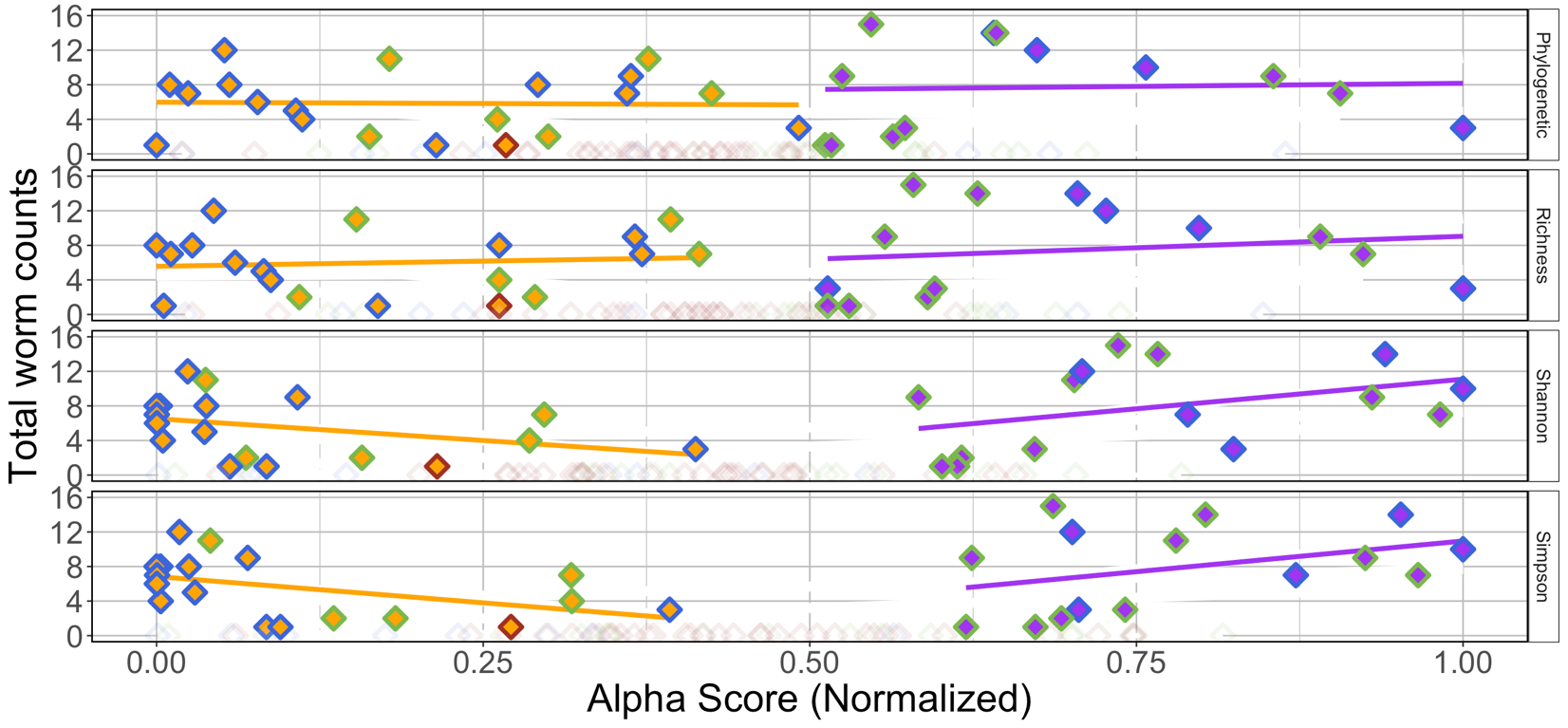 |
| --- |

## S5D)

### S5D.1)

| 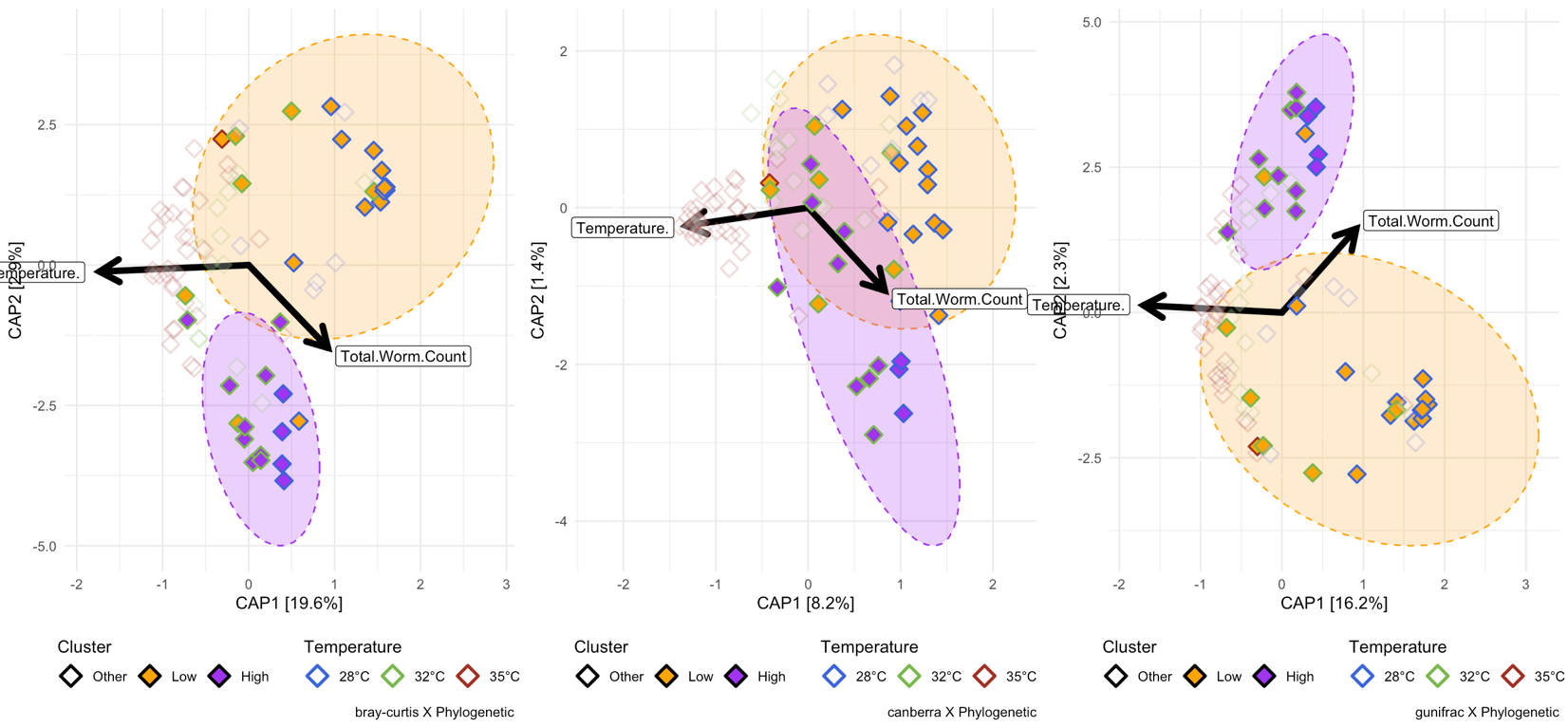 |
| --- |

### S5D.2)

| 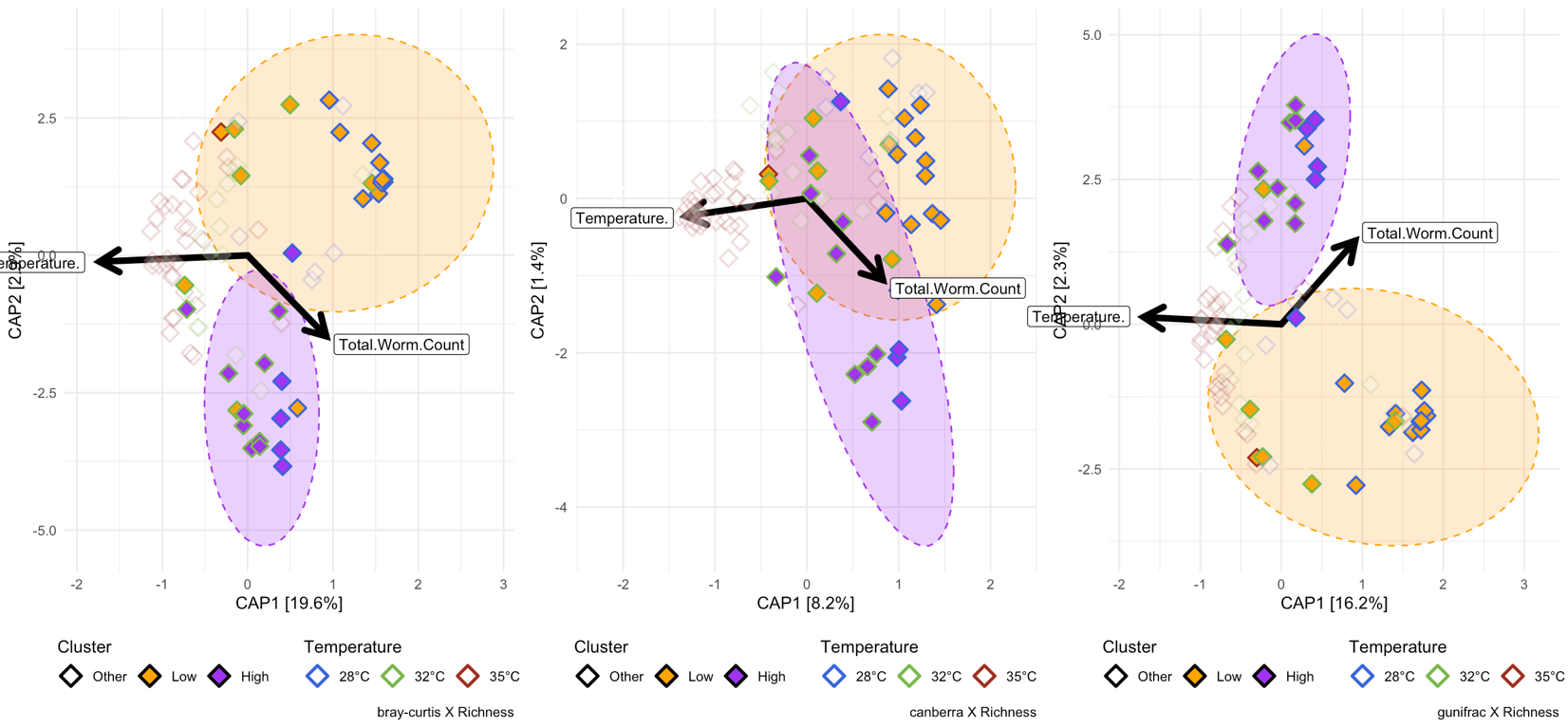 |
| --- |

### S5D.3)

| 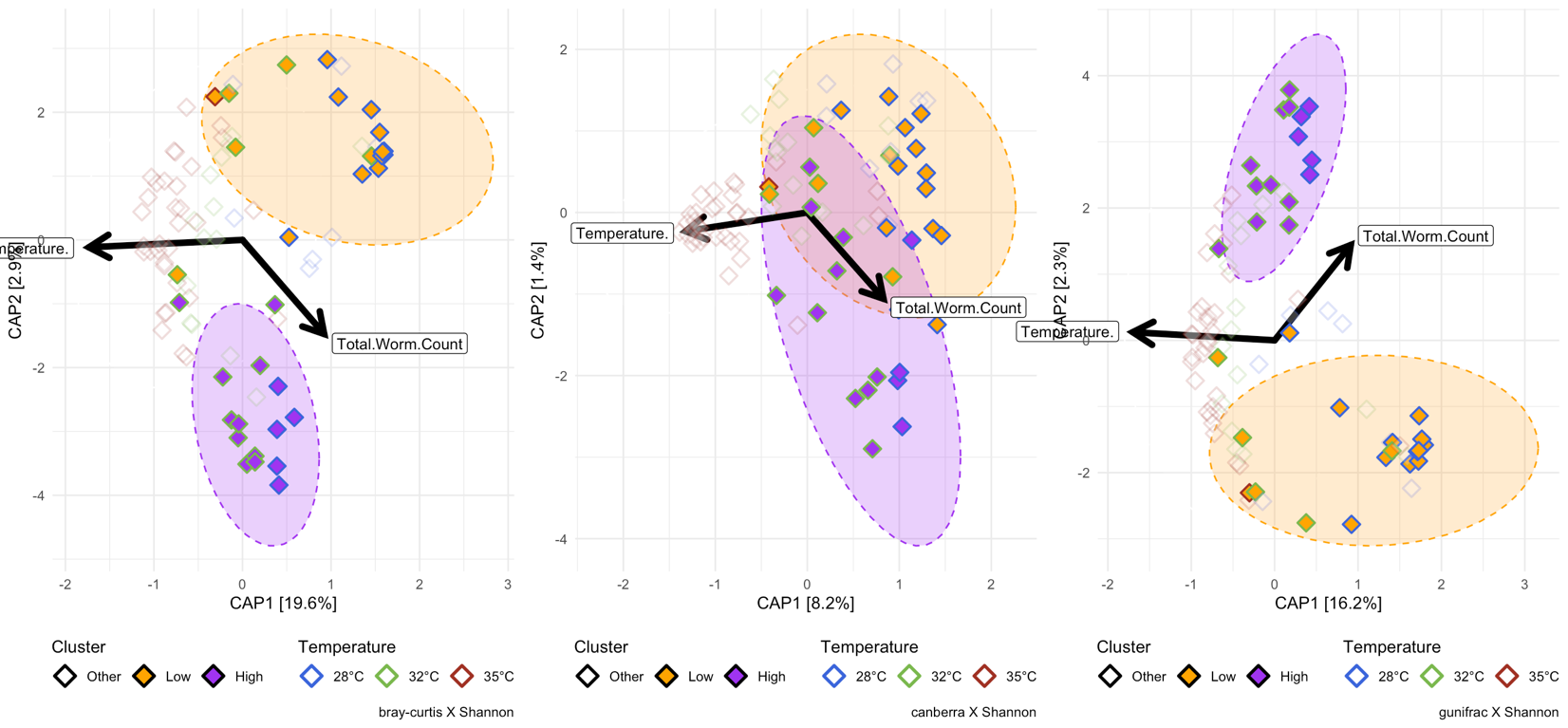 |
| --- |

### S5D.4)

| 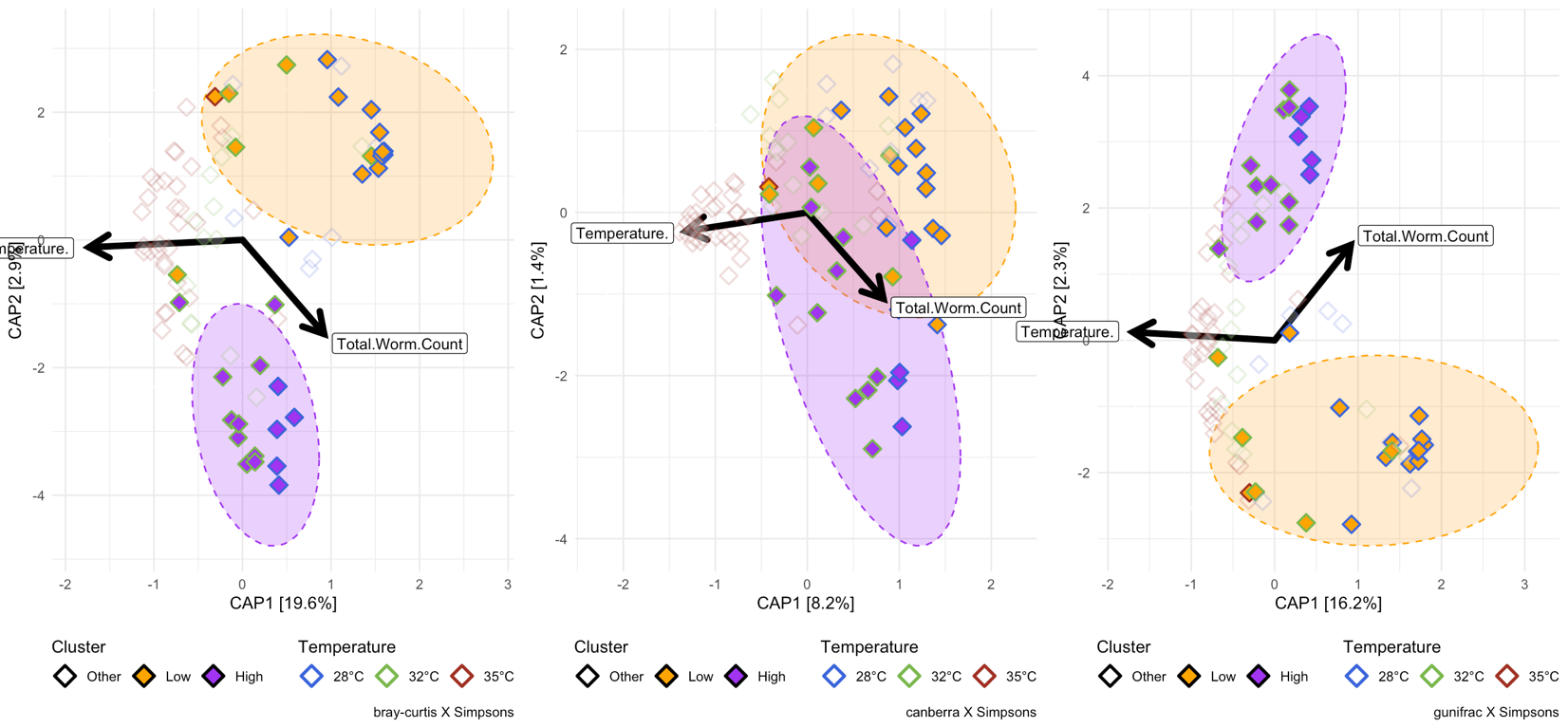 |
| --- |

# 6) Parasite exposure exacerbates water temperature differences in gut microbiome structure

## S6A)

| 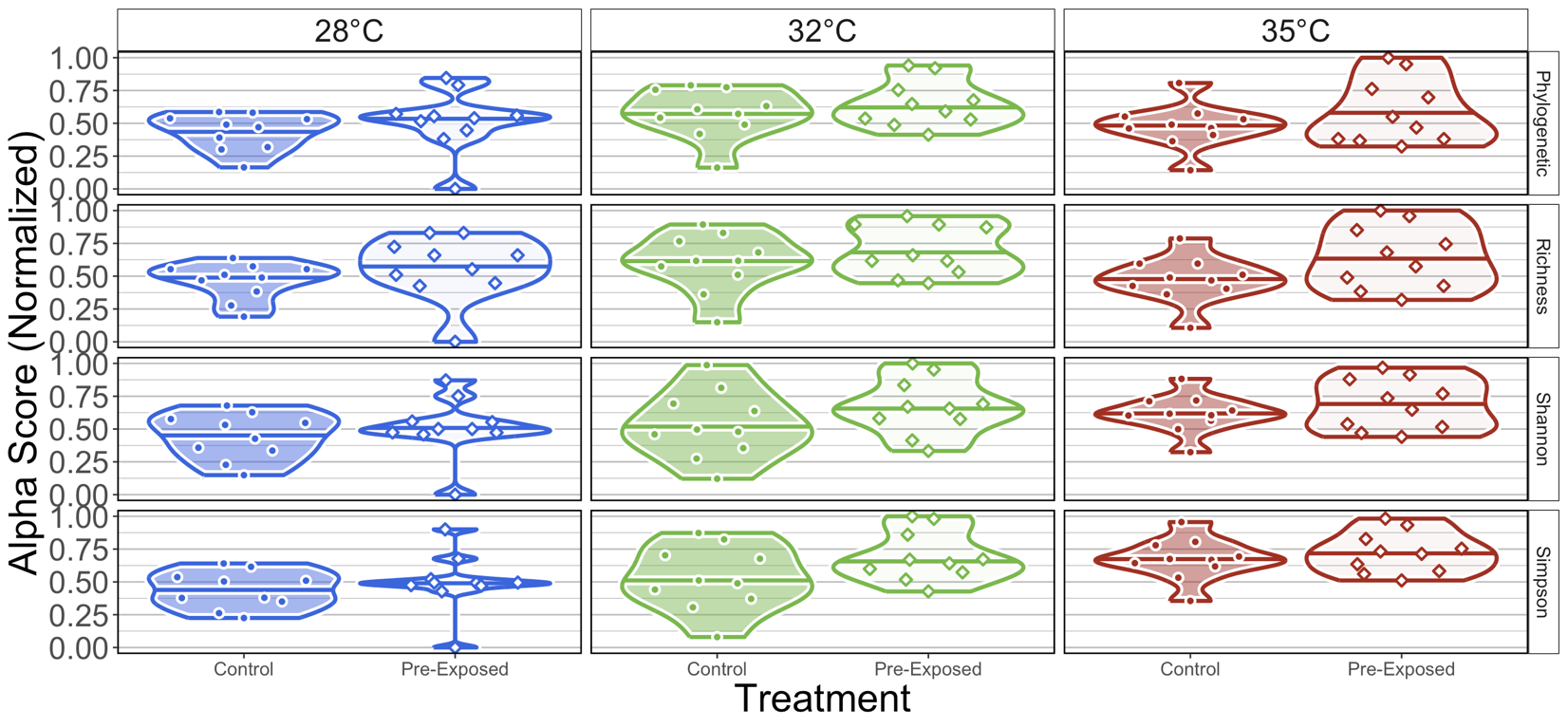 |
| --- |

## S6B)

| 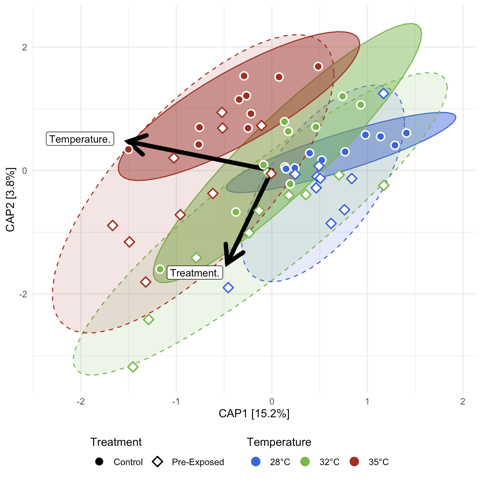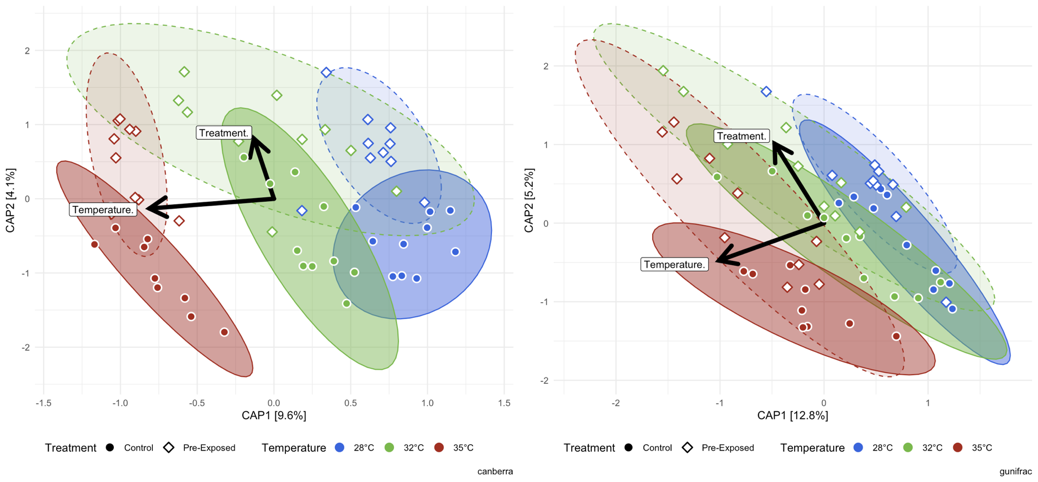 |
| --- |

## S6C)

| 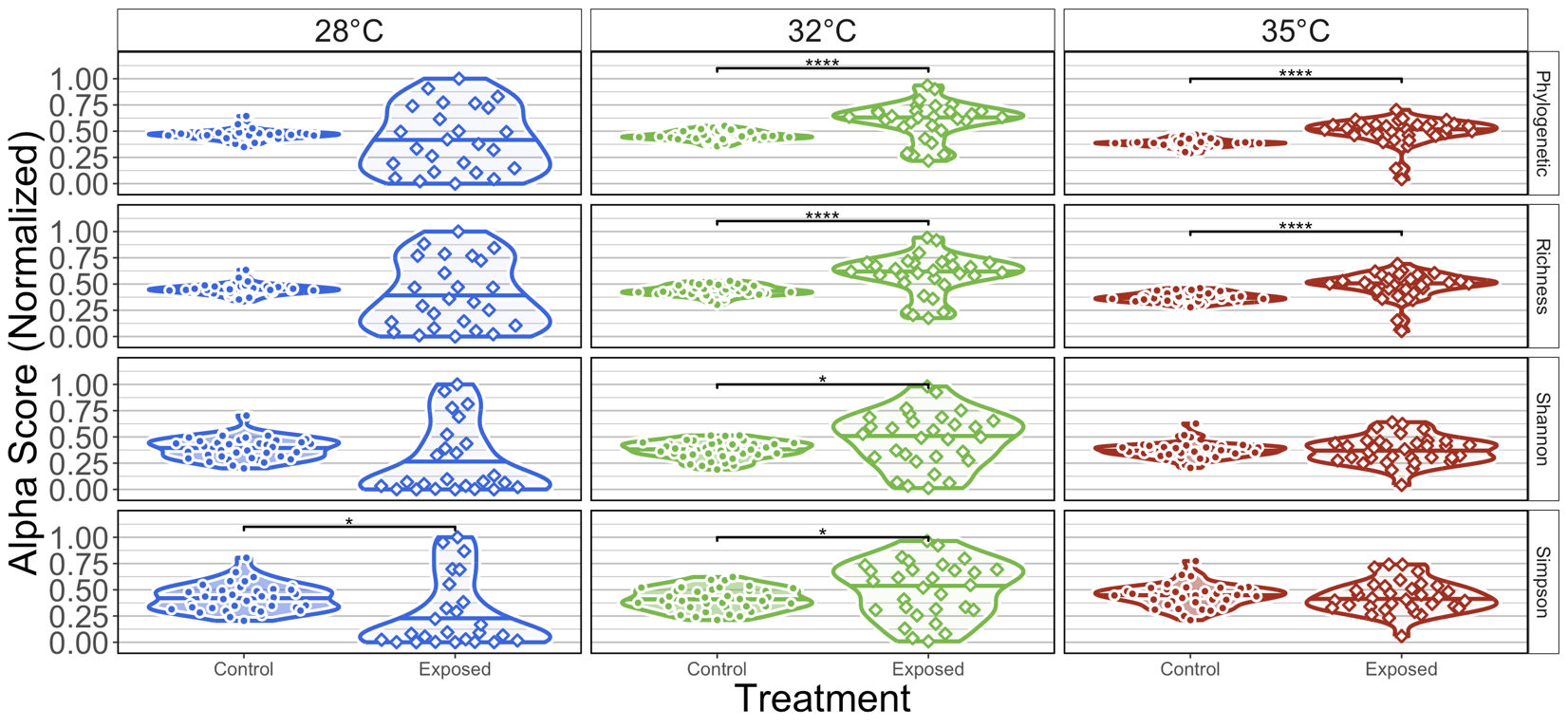 |
| --- |

## S6D)

| 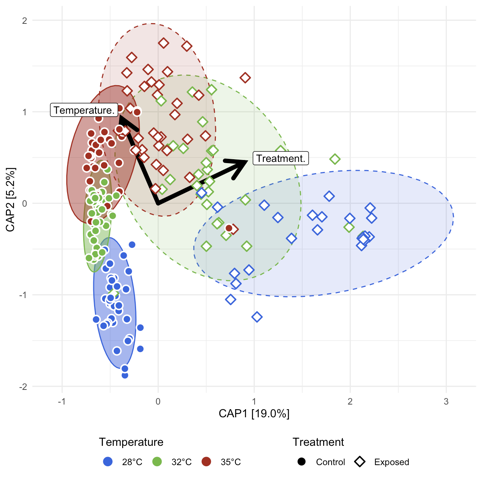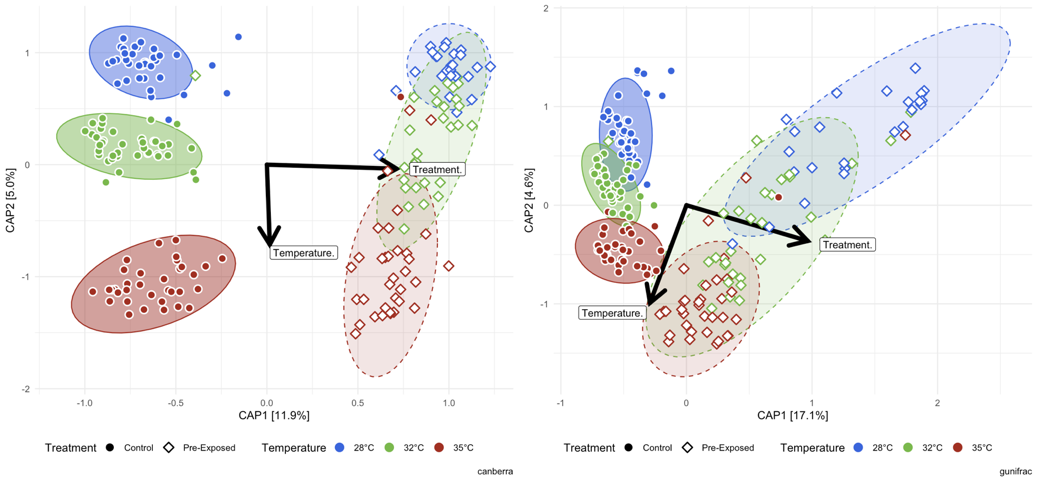 |
| --- |
